# Correction: Clinical improvement after surgery for degenerative cervical myelopathy; A comparison of Patient-Reported Outcome Measures during 12-month follow-up

**DOI:** 10.1371/journal.pone.0300610

**Published:** 2024-03-11

**Authors:** Christer Mjåset, John-Anker Zwart, Frode Kolstad, Tore Solberg, Margreth Grotle

The original ethics statement for this article [1] is incorrect.

The correct ethics statement is: This study’s research protocol (2014/1477) was reviewed by the Regional Committees for Medical and Health Research Ethics (REK) and was assessed to fall outside the scope of the Health Research Act. This means that the study did not require prior approval from the ethical committees.

The authors apologize for the error in the published article.
