# Supplementary material for: Clinical improvement after surgery for degenerative cervical myelopathy; A comparison of Patient-Reported Outcome Measures during 12-month follow-up
Source: PLoS One. 2022 Mar 8;17(3):e0264954. doi: 10.1371/journal.pone.0264954 (PMC8903279; doi:10.1371/journal.pone.0264954)
Supplement: S1 Table — Spearman, Spearman’s rank correlation coefficient; Neck Disability Index (0–100); SD, Standard Deviation; NRS-AP, Numeric Rating Scale for arm pain (0–10); NRS-NP, Numeric Rating Scale for neck pain (0–10), EQ-5D-3L, Health-Related Quality-of-Life by EuroQol (-0.4–1.0), EMS, European Myelopathy Score (5–18). (DOCX) [file pone.0264954.s001.docx]

**S1 Table:** Mean scores with standard deviation of the Patient-Reported Outcome Measures at 12 months for the whole myelopathy group according to the Global Perceived Effect Scale.

|  | | **Global Perceived Effect Scale** | | | | | | | |
| --- | --- | --- | --- | --- | --- | --- | --- | --- | --- |
|  |  | Completely  Recovered  (10.3%) | Much  Better  (36.0%) | Slightly  Better  (25.0%) | Unchanged  (12.9%) | Slightly worse  (9.4%) | Much worse  (5.6%) | Worse  than ever  (1.0%) | Spearman |
| **NDI**  **Change score** | Mean | 24.67 | 13.29 | 5.01 | 2.44 | 0.20 | -4.56 | -16.67 | 0.50 |
|  | SD | 14.48 | 14.52 | 11.82 | 11.35 | 17.31 | 14.22 | 9.52 |  |
| **NDI**  **Percentage**  **Change Score** | Mean | 78.27 | 35.40 | 4.42 | 3.69 | -24.23 | -62.31 | -95.20 | 0.58 |
|  | SD | 29.03 | 54.47 | 72.40 | 35.35 | 89.88 | 197.20 | 116.38 |  |
| **NRS-AP**  **Change score** | Mean | 3.79 | 2.20 | 1.00 | 0.76 | 0.70 | 0.00 | -2.50 | 0.31 |
|  | SD | 3.35 | 2.82 | 2.87 | 2.71 | 2.89 | 3.18 | 2.12 |  |
| **NRS-AP**  **Percentage**  **Change score** | Mean | 72.07 | 37.69 | 16.96 | -4.00 | -1.43 | -2.64 | -47.14 | 0.40 |
|  | SD | 43.04 | 72.46 | 61.47 | 80.36 | 75.25 | 62.43 | 46.47 |  |
| **NRS-NP**  **Change score** | Mean | 4.16 | 2.54 | 1.23 | 0.07 | 0.12 | -0.05 | 0.00 | 0.43 |
|  | SD | 3.27 | 2.58 | 2.79 | 2.82 | 2.33 | 1.96 | 1.41 |  |
| **NRS-NP**  **Percentage Change score** | Mean | 83.07 | 52.93 | 10.48 | -13.53 | -3.46 | -29.76 | 2.86 | 0.55 |
|  | SD | 35.20 | 41.71 | 75.95 | 91.19 | 46.94 | 103.26 | 24.24 |  |
| **EQ-5D-3L**  **Change score** | Mean | 0.34 | 0.22 | 0.13 | 0.07 | 0.00 | -0.07 | -0.20 | 0.38 |
|  | SD | 0.36 | 0.29 | 0.31 | 0.31 | 0.44 | 0.25 | 0.31 |  |
| **EQ-5D-3L**  **Percentage**  **Change score** | Mean | 21.52 | 13.72 | 8.29 | 4.36 | -0.01 | -4.55 | -12.38 | 0.38 |
|  | SD | 22.89 | 18.11 | 19.20 | 19.31 | 27.49 | 15.75 | 19.21 |  |
| **EMS**  **Change score** | Mean | 1.86 | 1.25 | 0.68 | 0.00 | 0.18 | -0.25 | -2.33 | 0.35 |
|  | SD | 1.95 | 1.91 | 1.86 | 1.34 | 1.70 | 1.48 | .58 |  |
| **EMS**  **Percentage**  **Change score** | Mean | 21.91 | 24.82 | 18.35 | 2.35 | 6.43 | -0.54 | -33.89 | 0.30 |
|  | SD | 29.83 | 84.50 | 79.61 | 21.42 | 32.13 | 18.58 | 22.99 |  |

Spearman, Spearman’s rank correlation coefficient; NDI, Neck Disability Index (0-100); SD, Standard Deviation; NRS-AP, Numeric Rating Scale for arm pain (0-10); NRS-NP, Numeric Rating Scale for neck pain (0-10); EQ-5D-3L, Health-Related Quality-of-Life by EuroQol (-0.4-1.0); EMS, European Myelopathy Score (5-18).
