# Supplementary material for: Clinical improvement after surgery for degenerative cervical myelopathy; A comparison of Patient-Reported Outcome Measures during 12-month follow-up
Source: PLoS One. 2022 Mar 8;17(3):e0264954. doi: 10.1371/journal.pone.0264954 (PMC8903279; doi:10.1371/journal.pone.0264954)
Supplement: S2 Table — NDI, Neck Disability Index (0–100); AUC, Area Under the Curve, NRS-AP, Numeric Rating Scale for arm pain (0–10), NRS-NP, Numeric Rating Scale for neck pain (0–10), EQ-5D-3L, Health-Related Quality-of-Life by EuroQol (-0.4–1.0); EMS, European Myelopathy Score (5–18). (DOCX) [file pone.0264954.s002.docx]

**S2 Table:** Area under the curve and cut-off estimates for “Minimal Clinically Important Difference” for all Patient-Reported Outcome Measures at 3 months**.**

|  | | **Change score**  **(points)** | **Percentage change score (%)** |
| --- | --- | --- | --- |
| **NDI** | **AUC**  **(95% CI)** | 0.81 (0.75, 0.87) | 0.81 (0.75, 0.88) |
|  | **Cut-off**  **(% sensitivity,**  **% specificity)** | 3.5 (0.76, 0. 74) | 14.6 (0.76, 0.76) |
| **NRS-AP** | **AUC**  **(95% CI)** | 0.76 (0.69, 0.83) | 0.78 (0.70, 0.86) |
|  | **Cut-off**  **(% sensitivity,**  **% specificity)** | 0.5 (0.78, 0.64) | 27.5 (0.75 0.73) |
| **NRS- NP** | **AUC**  **(95% CI)** | 0.71 (0.63, 0.78) | 0.70 (0.61, 0.78) |
|  | **Cut-off**  **(% sensitivity,**  **% specificity)** | 0.5 (0.67, 0.64) | 18.3 (0.71, 0.69) |
| **EQ-5D-3L** | **AUC**  **(95% CI)** | 0.74 (0.66, 0.82) | 0.73 (0.65, 0.82) |
|  | **Cut-off**  **(% sensitivity,**  **% specificity)** | 0.02 (0.70, 0.71) | 2.3 (0.67, 0.71) |
| **EMS** | **AUC**  **(95% CI)** | 0.72 (0.65, 0.80) | 0.69 (0.61, 0.78) |
|  | **Cut-off**  **(% sensitivity,**  **% specificity)** | 0.5 (0.65, 0.71) | 4.2 (0.65, 0.65) |

NDI, Neck Disability Index (0-100); AUC, Area Under the Curve; NRS-AP, Numeric Rating Scale for arm pain (0-10); NRS-NP, Numeric Rating Scale for neck pain (0-10); EQ-5D-3L, Health-Related Quality-of-Life by EuroQol (-0.4-1.0); EMS, European Myelopathy Score (5-18).
