# Supplementary material for: Clinical improvement after surgery for degenerative cervical myelopathy; A comparison of Patient-Reported Outcome Measures during 12-month follow-up
Source: PLoS One. 2022 Mar 8;17(3):e0264954. doi: 10.1371/journal.pone.0264954 (PMC8903279; doi:10.1371/journal.pone.0264954)
Supplement: S3 Table — NDI, Neck Disability Index (0–100), NRS-AP, Numeric Rating Scale for arm pain (0–10), NRS-NP, Numeric Rating Scale for neck pain (0–10), EQ-5D-3L, Health-Related Quality-of-Life by EuroQol (-0.4–1.0), EMS, European Myelopathy Score (5–18). (DOCX) [file pone.0264954.s003.docx]

**S3 Table:** Proportion of patients with an improvement larger than “Minimal Clinically Important Difference” at 12-months follow-up according to Patient-Reported Outcome Measures.

|  |  | Whole group  (%) | Anterior approach  (%) | Posterior approach  (%) |
| --- | --- | --- | --- | --- |
| **NDI** | Change score  % change score | 58  59 | 61  62 | 57  57 |
| **NRS-AP** | Change score  % change score | 61  56 | 62  58 | 59  56 |
| **NRS-NP** | Change score  % change score | 62  61 | 67  63 | 54  58 |
| **Eq-5D-3L** | Change score  % change score | 60  59 | 62  60 | 57  53 |
| **EMS** | Change score  % change score | 51  51 | 53  53 | 49  49 |

NDI, Neck Disability Index (0-100); NRS-AP, Numeric Rating Scale for arm pain (0-10); NRS-NP, Numeric Rating Scale for neck pain (0-10); EQ-5D-3L, Health-Related Quality-of-Life by EuroQol (-0.4-1.0); EMS, European Myelopathy Score (5-18).
